# Spectroscopic and Theoretical Study of the Intramolecular π-Type Hydrogen Bonding and Conformations of 2-Cyclopenten-1-ol

**DOI:** 10.3390/molecules26041106

**Published:** 2021-02-19

**Authors:** Esther J. Ocola, Jaan Laane

**Affiliations:** 1 Department of Chemistry, Texas A&M University, College Station, TX 77843-3255, USA; eocola@chem.tamu.edu; 2 Institute for Quantum Science and Engineering, Texas A&M University, College Station, TX 77843-4242, USA

**Keywords:** 2-cyclopenten-1-ol, π-type intramolecular hydrogen bonding, infrared spectroscopy, conformations, potential energy surface, theoretical calculations

## Abstract

The conformations of 2-cyclopenten-1-ol (2CPOL) have been investigated by high-level theoretical computations and infrared spectroscopy. The six conformational minima correspond to specific values of the ring-puckering and OH internal rotation coordinates. The conformation with the lowest energy possesses intramolecular π-type hydrogen bonding. A second conformer with weaker hydrogen bonding has somewhat higher energy. Ab initio coupled-cluster theory with single and double excitations (CCSD) was used with the cc-pVTZ (triple-ζ) basis set to calculate the two-dimensional potential energy surface (PES) governing the conformational dynamics along the ring-puckering and internal rotation coordinates. The two conformers with the hydrogen bonding lie about 300 cm^−1^ (0.8 kcal/mole) lower in energy than the other four conformers. The lowest energy conformation has a calculated distance of 2.68 Å from the hydrogen atom on the OH group to the middle of the C=C double bond. For the other conformers, this distance is at least 0.3 Å longer. The infrared spectrum in the O-H stretching region agrees well with the predicted frequency differences between the conformers and shows the conformers with the hydrogen bonding to have the lowest values. The infrared spectra in other regions arise mostly from the two hydrogen-bonded species.

## 1. Introduction

**Scheme 1:**
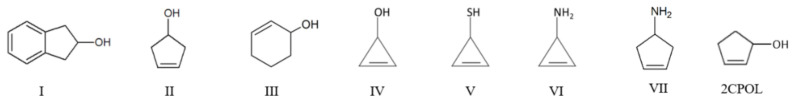
Molecules with intramolecular π-type hydrogen bonding.

For many years, our research group has focused on utilizing both experiment and theory to develop a more comprehensive understanding of one-dimensional vibrational potential energy functions (PEFs) and two-dimensional potential energy surfaces (PESs) [1,2,3,4,5,6,7]. In particular, we have investigated the PEFs and PESs which govern the conformational dynamics of small ring molecules as well as those governing internal rotations. In recent years, we have applied our expertise to investigating cyclic molecules that possess intramolecular π-type hydrogen bonding. Among the molecules which we studied are 2-indanol (I) [8], 3-cyclopenten-1-ol (II) [9,10], 2-cyclohexen-1-ol (III) [11], 2-cyclopropen-1-ol (IV) [12], 2-cyclopropen-1-thiol (V) [12], 2-cyclopropen-1-amine (VI) [12], and 3-cyclopenten-1-amine (VII) [13]. These molecules are shown in Scheme 1.

Each of these molecules can exist as several different conformers which are determined by specific coordinate values of the internal rotations of the OH groups and/or the out-of-plane ring bending modes (ring-puckering or ring-twisting). For each of these molecules, the conformer with the intramolecular π-type hydrogen bonding has the lowest energy, and the π bonding stabilizations range from about 2 to 10 kJ/mol. In our present study, we focus our attention on the π-type hydrogen bonding of the OH group to the C=C double bonds of 2-cyclopenten-1-ol (2CPOL).

Other researchers have also reported previous experimental evidence of intramolecular π-type hydrogen bonding between OH groups and C=C double bonds in molecules such as 2-allylphenol [14], 3-buten-2-ol [15], allyl alcohol [16], 1,4-pentadien-3-ol [17], 2-cyclopropylideneethanol [18], 3-buten-1-ol [19], 1-ethenylcyclopropan-1-ol [20], 4-substituted 2-allylphenols [21], allyl-carbinol [22,23], and methallyl-carbinol [23].

In this paper, we report our experimental infrared spectra and computational results for 2CPOL. The focus of the work was to investigate the different conformations of this molecule and to understand the nature of the π-type hydrogen bond involving the OH group. In addition, we wished to investigate the two-dimensional potential energy surface (PES) that governs the conformational changes.

## 2. Results and Discussion

### 2.1. Calculated Molecular Conformations

**Figure 1:**
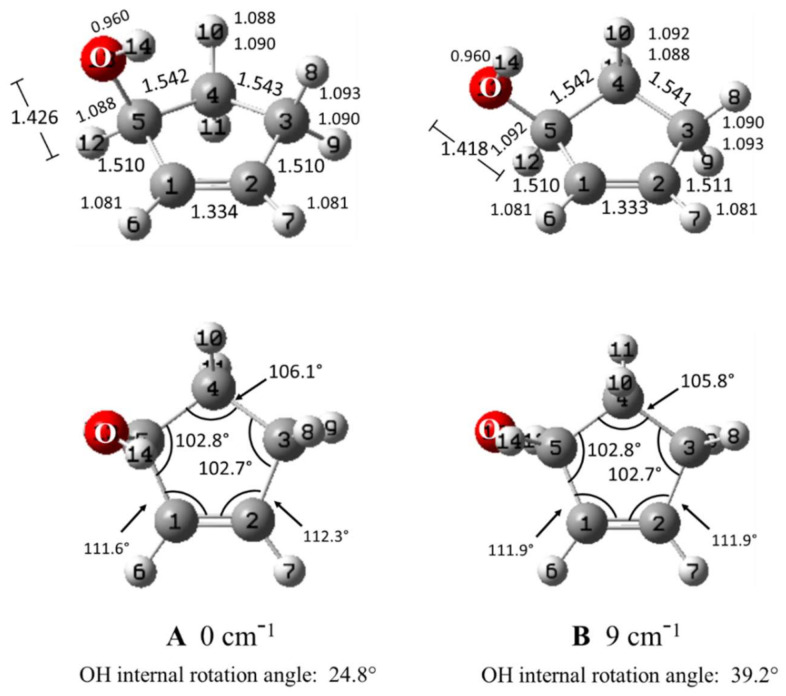
Calculated structures of the lowest energy conformers of 2CPOL from coupled-cluster theory with single and double excitations (CCSD)-pVTZ computations.

**Figure 2:**
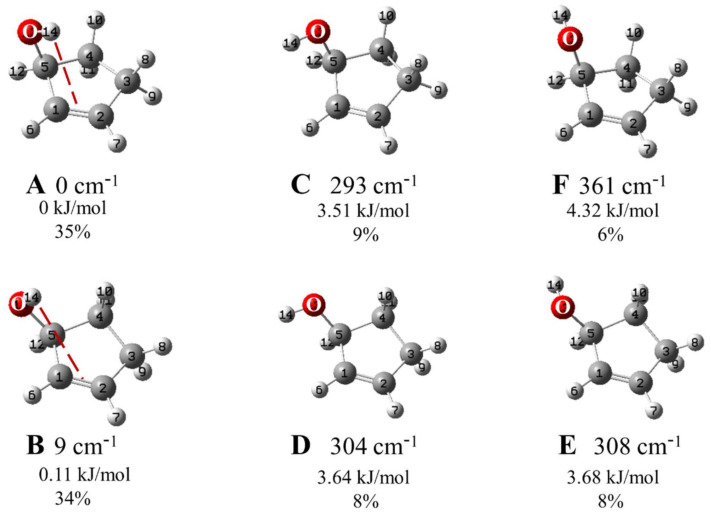
Calculated conformational minima, relative energies, and calculated abundances at 25 °C of 2CPOL from CCSD/cc-pVTZ computations. Conformers A, C, and F are puckered down, whereas conformers B, D, and E are puckered up.

The six conformers of 2CPOL with energy minima on the PES result from different values of the ring-puckering coordinate and the internal rotation angle of the OH group. Table 1 presents the key features of these conformations. Figure 1 shows the atomic numbering and the geometrical parameters calculated for the two lowest energy conformers. The lowest energy conformation A clearly possesses π-type hydrogen bonding as the calculated distance from the hydrogen atom on the OH group to the middle of the C=C double bond is 2.68 Å. This is very similar to the distance of 2.75 Å for 3-cyclopenten-1ol (II) and for other cyclic alcohols with this type of π bonding as shown in Table 2. The conformers A and B differ by having carbon atom 4 puckered in opposite directions. According to the CCSD/cc-pVTZ computations, B is only 9 cm^−1^ higher in energy from A, whereas the other conformers are 293 to 361 cm^−1^ higher in energy. MP2/cc-pVTZ computations predict B to be 89 cm^−1^ higher in energy. For B, the calculated distance from the hydrogen atom on the OH group to the middle of the C=C double bond is 3.00 Å, and this is considerably longer than that in the other molecules shown in Table 2, suggesting that the π type hydrogen bonding, if present, is quite small. On the other hand, as discussed below, the calculated and observed O-H stretching frequencies for A and B are both considerably lower than for the other conformers, and this would result from the hydrogen bonding. Figure 2 shows all six calculated structures for the conformational minima. Table 1 presents the ring-puckering angles and OH internal rotation angles, the calculated energy differences, and the calculated relative populations of the six conformers of 2CPOL at 25 °C. Supplementary Table S1 presents the calculated structural parameters for all six conformers from our CCSD/cc-pVTZ computations. Table 1 and Table S1 also show the calculated distances from the hydrogen atoms of the OH group to the center of the C=C bond.

As can be seen in Table 1, the absolute value of the ring-puckering angle is 22° ± 2° for each of the conformers. Thus, the conformational energy differences arise primarily from the internal rotation of the OH group. Conformers A and B differ by about 14° of the OH internal rotational angle, and both may allow the intramolecular hydrogen bonding to occur. However, the A conformer is lower in energy and has a stronger hydrogen bond as reflected by the shorter distance from the OH hydrogen to the center of the C=C bond. For conformer A, this distance is 2.682 Å, whereas for the conformers without hydrogen bonding, the distances are at least 0.3 Å longer.

### 2.2. Vibrational Spectra

**Figure 3:**
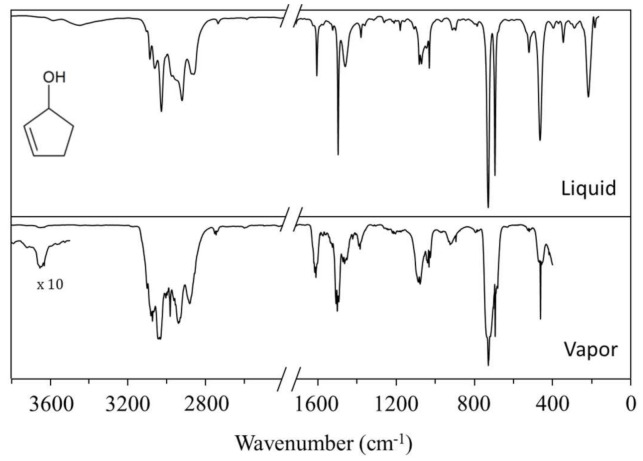
Observed infrared spectra of 2CPOL.

Figure 3 shows a comparison of the experimental liquid phase and vapor-phase infrared spectra. Since intermolecular hydrogen bonding will be much stronger in the liquid phase than the intramolecular hydrogen bonding, bands corresponding to the specific A, B, C, D, E, and F conformers will not be present in the liquid spectra. Table 3 shows the observed vapor-phase and calculated frequencies for selected vibrations of the A conformer of 2CPOL compared to those observed for the related molecules 3-cyclopentenol (II) [9], 3-cyclopenten-1-amine (VII) [13], and cyclopentene [24].

**Figure 4:**
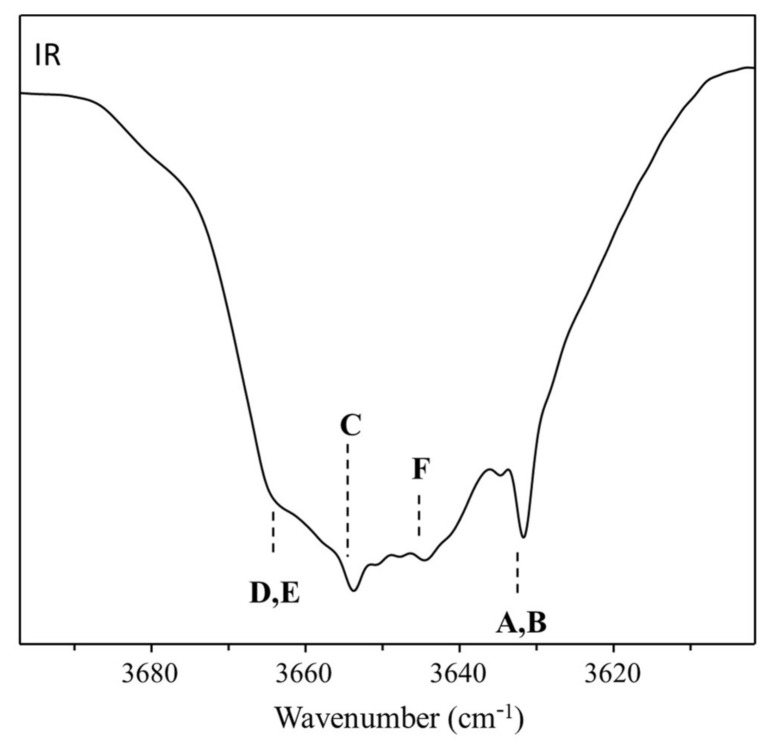
Observed vapor-phase infrared of 2CPOL for the O-H stretch region.

**Figure 5:**
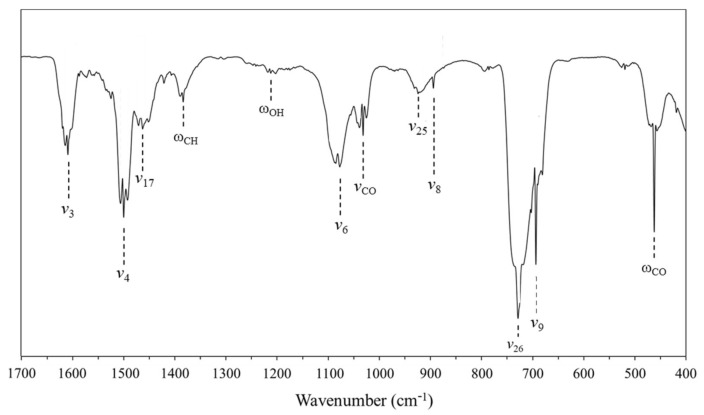
Observed vapor-phase infrared of 2CPOL for the 400–1700 cm^−1^ region.

Figure 4 shows the observed infrared band for the conformers of 2CPOL for the O-H stretch region. Based on the calculated frequency differences for the different conformers, the observed IR band at 3632 cm^−1^ is assigned to both A and B; 3654 cm^−1^ is assigned to C; 3664 cm^−1^ is assigned to D; 3664 cm^−1^ is assigned to E; and 3644 cm^−1^ is assigned to F. Table 4 shows the observed O-H stretching frequencies for the six conformers of 2CPOL compared to the calculated values. As expected, the weak hydrogen bonding lowers the O-H stretching frequency so that conformers A and B have the lowest value. The weakening of the O-H bond due to its interaction with the C=C double bond is supported by our CCSD/cc-pVTZ computations. The good agreement between observed and calculated frequencies strongly supports the presence of the six expected conformers. Figure 5 shows the vibrational spectra and assignments of 2CPOL in the 400–1700 cm^−1^ region. Due to the relatively low molecular populations of the other conformers, all of the bands have been assigned to A and B, which make up 69% of the molecular population. The A and B frequencies are predicted to differ very little in this region so each of the bands seen in Figure 5 arises mainly from both conformers.

### 2.3. Potential Energy Surface

**Figure 6:**
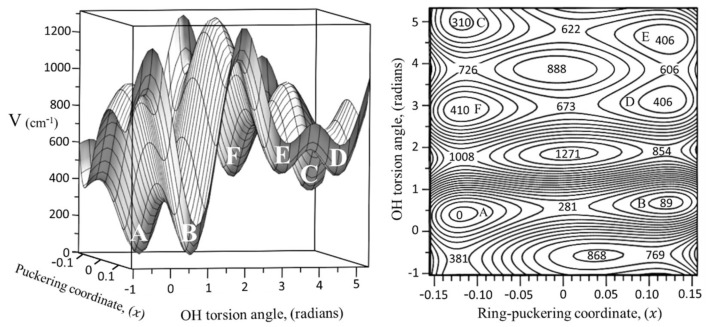
Potential energy surface (PES) and contour map of 2CPOL in terms of its ring-puckering and the OH internal rotation coordinates.

Figure 6 shows the calculated potential energy surface (PES) for 2CPOL in terms of its ring-puckering and OH internal rotation coordinates. This PES was generated by individually computing the energies of more than 150 conformations of 2CPOL using MP2/cc-pVTZ calculations. CCSD/cc-pVTZ calculations for so many conformations would have taken an inordinate amount of computer time. The zero value of the OH internal rotation angle is defined at the position where the hydrogen atom of the hydroxyl group OH points toward the center of the C=C double bond. The PES clearly shows the presence of the six different minima which reside at six different conformational energies. The mathematical function that best satisfies the fit of the calculated data has the form:

(1) $$V\;=\;\overset{6}{\underset{i\;=\;i}{\sum }}a_{i}x^{i}+\;\overset{6}{\underset{n\;=\;1}{\sum }}b_{n}\sin\left(n\phi \right)+\overset{6}{\underset{n\;=\;1}{\sum }}c_{n}\sin\left(n\phi \right)\cos\left(n\phi \right)+\overset{3}{\underset{1}{\sum }}d_{n}\cos\left(n\phi \right)+x\left(\overset{6}{\underset{n\;=\;1}{\sum }}e_{n}\sin\left(n\phi \right)+\overset{6}{\underset{n\;=\;1}{\sum }}f_{n}\cos\left(n\phi \right)\right)$$

where *x* and *y* refer to the puckering and internal rotation coordinates. This equation consists of a polynomial component dependent on the x term to describe the ring-puckering motion, three sums of trigonometric functions to define the periodicity of the OH internal rotation, and a final set of cross terms, which describe the interaction between the ring-puckering and the internal rotation. Equation (1) is complex due to the lack of symmetry in the 2CPOL molecule. Figure 6 also shows the contour map of the PES calculated for 2CPOL. The calculated interconversion barriers from the MP2/cc-pVTZ computations are also indicated. Table 5 shows a comparison of the calculated results for 2CPOL from CCSD/cc-pVTZ computations to those from MP2/cc-pVTZ computations. We expect the CCSD calculations to be more reliable, but it is gratifying to see that the differences are relatively small.

The PES shows the barrier to ring planarity to be 281 cm^−1^ and the internal rotation barriers between energy minima to be in the 300 to 1000 cm^−1^ range.

## 3. Materials and Methods

2CPOL (95% purity) was purchased from CHEMSAMPCo and was purified by trap-to-trap distillation. Infrared spectra with a spectral resolution of 0.5 cm^−1^ were recorded using a Bruker Vertex 70 instrument, which was purged by a stream of dry nitrogen gas. The mid-infrared vapor-phase spectrum of 2CPOL was obtained at its vapor pressure at room temperature in a 10 cm cell. 2CPOL has a boiling point of 137 °C and vapor pressure of 3.1 Torr at 25 °C. KBr windows were used for the infrared cell. Spectra of liquid samples were recorded by placing a drop between either KBr plates for the mid-infrared or between CsI plates for the far-infrared. We were not able to record the vapor-phase Raman spectrum of this molecule at the higher temperatures required to obtain sufficient vapor pressure.

### 3.1. Computations

#### 3.1.1. Structure and Frequency Calculations

The ab initio coupled-cluster theory with single and double excitations (CCSD) method was used with the cc-pVTZ (triple-ζ) basis set to calculate the conformational energies and to compute the geometries for each of the six conformers of 2CPOL. The GAUSSIAN 16 program [25] was used for the computations, and the GaussView 6 program [26] was used to visualize the structures.

Vibrational frequencies were calculated using MP2/cc-pVTZ computations. The scaling factors used for the MP2/cc-pVTZ calculations were 0.970 for frequencies below 1600 cm^−1^, 0.976 for 1600–2000 cm^−1^, 0.976 for 1600–2000 cm^−1^, and 0.948 for higher frequencies except for the O-H stretching region [13]. For the O-H region, 3520–3680 cm^−1^ the scaling factor of 0.950 was chosen to match the observed value for the lowest energy hydrogen-bonded conformer so that the predicted frequency shifts could readily be calculated.

#### 3.1.2. Potential Energy Surface

**Figure 7:**
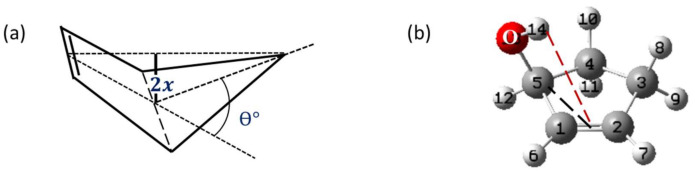
(**a**) Definition of the ring-puckering coordinate (x), where 2x is the distance between the two dotted lines and Ө is the ring puckering angle. (**b**) 2-cyclopenten-1-ol (2CPOL).

The data points required for determining the theoretical PES of 2CPOL and its contour map were obtained from MP2/cc-pVTZ computations. The MAPLE 2015.1 computing environment [27] was used to perform the mathematical fit and plot the data. The PES was generated in terms of the ring-puckering coordinate (x), which is defined in Figure 7a, and the OH internal rotational angle. This angle is defined as the dihedral angle between the OH bond and the black dotted line that connects atom 5 to the mid-point of the C=C double bond as shown in Figure 7b.

## 4. Conclusions

2CPOL can exist in six different conformers and the one with the lowest energy shows π-type hydrogen bonding. This conformation has an OH hydrogen to the center of the C=C bond distance of 2.68 Å. This value is similar to those we found previously for the related molecules II to IV. The PES for 2CPOL derived in this work shows how the six conformers of this molecule can interconvert from one to another and what are the magnitudes of the barriers that must be overcome. It also shows that the intramolecular π-type hydrogen bonding lowers the conformational energy by approximately 300 cm^−1^ (0.8 kcal/mole). The experimental infrared spectrum in the O-H stretching region shows evidence for the presence of the predicted conformers.

## Figures and Tables

**Table 1:** Selected characteristics from CCSD/cc-pVTZ computations for all of the six 2CPOL conformers.

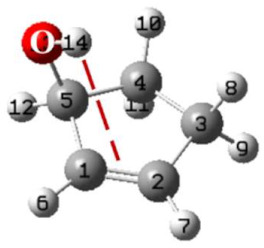	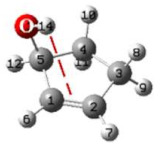	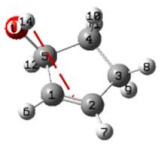	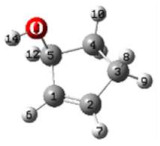	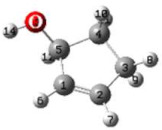	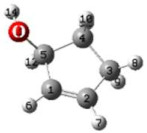	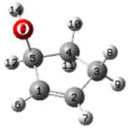
	**2CPOL Conformers**					
	**A**	**B**	**C**	**D**	**E**	**F**
Energy						
Energy (cm^−1^)	0	9	293	304	308	361
Energy (kJ/mol)	0	0.11	3.51	3.64	3.68	4.32
Energy (kcal/mol)	0	0.03	0.84	0.87	0.88	1.03
Relative population (%)						
At 25°	35%	34%	9%	8%	8%	6%
Angles (degrees)						
Ring-puckering angle	−21.1°	22.5°	−23.1°	22.8°	23.7°	−22.8°
OH internal rotation angle, ϕ	24.8°	39.2°	−75.7°	−91.0°	172.3°	166.9°
Distance (Å)						
O−H_14_	0.960	0.960	0.958	0.958	0.958	0.958
dH_14_^a^	2.682	3.001	3.019	3.335	3.680	3.554

^a^ Distance between H_14_ of the OH group to the midpoint of the double C=C bond.

**Table 2:** Calculated H-π (C=C) bonding distances in cyclic molecules.

Molecule	H﮲﮲﮲π (C=C) Distance, Å	Method	Reference
2-indanol (I)	2.650	CCSD/cc-pVTZ	This work
	2.580	MP2/cc-pVTZ	This work
3-cyclopenten-1-ol (II)	2.744	CCSD/cc-pVTZ	This work
	2.673	MP2/cc-pVTZ	This work
2-cyclohexen-1-ol (III)	2.756	CCSD/cc-pVTZ	This work
	2.737	MP2/cc-pVTZ	[11]
2-cyclopropen-1-ol (IV)	2.488	CCSD/cc-pVTZ	[12]
	2.478	MP2/cc-pVTZ	[12]
2-cyclopropen-1-thiol (V)	2.774	CCSD/cc-pVTZ	[12]
	2.740	MP2/cc-pVTZ	[12]
2-cyclopropen-1-amine (VI)	2.583	CCSD/cc-pVTZ	[12]
	2.571	MP2/cc-pVTZ	[12]
3-cyclopenten-1-amine (VII)	2.850	CCSD/cc-pVTZ	[13]
	2.773	MP2/cc-pVTZ	This work
2-cyclopenten-1-ol (2CPOL)	2.682	CCSD/cc-pVTZ	This work
	2.632	MP2/cc-pVTZ	This work

**Table 3:** Observed and calculated frequency differences for selected vibrations conformer **A** of 2CPOL compared to those observed for other five-membered ring molecules.

		2-Cyclopenten-1-ol				3-Cyclopenten-1-ol		3-Cyclopenten-1-amine		Cyclopentene	
		Observed		Calculated		Observed					
		A		A		A		A		Puckered, *C*_s_	
Ring-Pseudo C_2v_		Freq.	IR ^a^	Freq.	(IR, R) ^b^	Freq. ^c^	(IR, R) ^d,e^	Freq. ^f^	(IR, R) ^d,f^	Freq. ^e^	(IR, R) ^b,e^
A1											
*v*_3_	C=C stretch	1609	m	1609	(3, 80)	1607	(2, 46)	1613	(m, 88)	1623	(m, 91)
*v*_4_	CH_2_ deformation	1500	s	1459	(8, 67)	1446^f^	(3, 19)	1452	(m, 17)	1471^f^	(vw, 16)
*v*_6_	=C-H in plane wag	1077	m	1095	(10, 100)	1108^f^	(4, 95)	1109	(w, 58)	1101	(w, 66)
*v*_8_	Ring stretch	894	m	869	(13, 60)	832	(5, 3)	804	(s, 43)	900	(m, 100)
*v*_9_	Ring angle bend	694	s	692	(6, 9)	745	(51, 77)	735	(w, 9)	796^g^	(vw, 1)
B1											
*v*_17_	CH_2_ deformation	1463	m	1436	(7, 77)	1446^f^	(17, 19)	1437	(m, 4)	1445^f^	(m, 23)
B2											
*v*_25_	CH_2_ rock	924	m	971	(14, 36)	948^f^	(29, 17)	934	(m, 4)	1047	(s, 1)
*v*_26_	=C-H out-of-plane wag	726	s	719	(20, 15)	674	(97, 12)	671	(s, 11)	695	(s, 1)
*v*_27_	Ring puckering	---	---	119	(2, 11)	---	(---, ---)	---	(---, ---)	---	(---, ---)
Other vibrations											
$v_{\mathrm{OH}}$	OH stretch	3632	w	3632	(32, 26)	3623	(41, 9)	N. A.	N. A.	N. A.	N. A.
$\omega_{\mathrm{CH}}$	C-H wag (up and down)	1385	m	1386	(80, 53)	1395	(38, 13)	1379	(m, 7)	N. A.	N. A.
$\omega_{\mathrm{OH}}$	COH wag	1212	w	1224	(13, 14)	1275	(9, 3)	N. A.	N. A.	N. A.	N. A.
${\;\omega }_{\mathrm{CO}}$	C-O wag (up and down)	462	s	487	(5, 21)	---	---	N. A.	N. A.	N. A.	N. A.
$\omega \prime_{\mathrm{CO}}$	C-O wag (sideways)	346 (L)	w	388	(12, 7)	444	(0.5, 2)	N. A.	N. A.	N. A.	N. A.
*v*_CO_	C-O stretch	1032	m	1045	(75, 21)	1048	(54, 0.7)	N. A.	N. A.	N. A.	N. A.
$\;\;\tau_{\mathrm{OH}}$	OH torsion	217 (L)	m	313	(100, 21)	397^h^	(---, 8)	N. A.	N. A.	N. A.	N. A.

^a^ IR intensities. s-strong, m-medium, w-weak. ^b^ Relative IR and Raman intensities. ^c^ From Reference [9], unless indicated. ^d^ From Reference [13], unless indicated. ^e^ From Reference [24], unless indicated. ^f^ Reassigned according to MP2/cc-pVTZ computations.

**Table 4:** O-H stretching frequencies (cm^−1^) for the six conformers of 2CPOL.

Conformer	Observed		Calculated	
	Frequency	Shift	Frequency	Shift
**A**	3632	0	3632	0
**B**	3632	0	3634	2
**C**	3654	22	3648	16
**D**	3664	32	3658	26
**E**	3664	32	3656	24
**F**	3644	12	3644	12

**Table 5:** Comparison of calculated conformational energies and geometrical parameters for 2CPOL from CCSD/cc-pVTZ and MP2/cc-pVTZ computations.

	2CPOL Conformers					
	A	B	C	D	E	F
Energy, (cm^−1^)						
CCSD/cc-pVTZ	0	9	293	304	308	361
MP2/cc-pVTZ	0	89	308	406	406	409
Ring-puckering coordinate, (Å)						
CCSD/cc-pVTZ	−0.102	0.109	−0.112	0.110	0.115	−0.110
MP2/cc-pVTZ	−0.114	0.118	−0.124	0.118	0.124	−0.122
Ring-puckering angle, (degrees)						
CCSD/cc-pVTZ	−21.1°	22.5°	−23.1°	22.8°	23.7°	−22.8°
MP2/cc-pVTZ	−23.6°	24.3°	−25.7°	24.4°	25.6°	−25.3°
OH internal rotation angle, (degrees)						
CCSD/cc-pVTZ	24.8°	39.2°	284.3°	269.0°	172.3°	166.9°
MP2/cc-pVTZ	22.6°	38.7°	286.4°	268.6°	172.3°	166.6°

## Data Availability

The data presented in this study are available in this article.

## Supplementary Materials

The following is available online, Table S1: Structural parameters for the six conformers of 2CPOL from CCSD/cc-pVTZ computations.

## Sample Availability

Not available.
