# Supplementary material for: Spectroscopic and Theoretical Study of the Intramolecular π-Type Hydrogen Bonding and Conformations of 2-Cyclopenten-1-ol
Source: Molecules. 2021 Feb 19;26(4):1106. doi: 10.3390/molecules26041106 (PMC7923001; doi:10.3390/molecules26041106)
Supplement: Supplementary file 1 [file molecules-26-01106-s001.pdf]

Spectroscopic and Theoretical Study of the Intramolecular π-Type Hydrogen Bonding and Conformations of 2-Cyclopenten-1-ol

Esther J. Ocola and Jaan Laane*

Department of Chemistry, Texas A&M University, College Station, TX 77843-3255, USA

Institute for Quantum Science and Engineering, Texas A&M University, College Station, 77843-4242, USA

Supplementary Material

(This file includes one table in three pages.)

Table of Contents

	Pages
Table S1. Structural parameters for the six conformers of 2CPOL from CCSD/cc-pVTZ computations.	S2-S3

Corresponding Author, Email address: laane@chem.tamu.edu, Phone: 979-845-3352

Table S1. Structural parameters for the six conformers of 2CPOL from CCSD/cc-pVTZ computations.

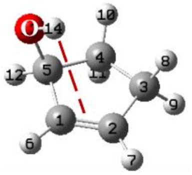

	2CPOL Conformer					
	**A**	**B**	**C**	**D**	**E**	**F**
**Bond lengths (Å)**						
C_1=C_2	1.334	1.333	1.333	1.333	1.333	1.333
C_1–C_5	1.510	1.510	1.512	1.511	1.505	1.505
C_2–C_3	1.510	1.511	1.509	1.511	1.511	1.511
C_3–C_4	1.543	1.541	1.542	1.541	1.542	1.542
C_4–C_5	1.542	1.542	1.533	1.534	1.541	1.541
C_1–H_6	1.081	1.081	1.082	1.082	1.080	1.080
C_2–H_7	1.081	1.081	1.081	1.081	1.081	1.081
C_3–H_8	1.093	1.090	1.093	1.090	1.090	1.090
C_3–H_9	1.090	1.093	1.090	1.093	1.094	1.094
C_4–H_{10}	1.088	1.092	1.088	1.090	1.091	1.091
C_4–H_{11}	1.090	1.088	1.090	1.088	1.090	1.090
C_5–H_{12}	1.088	1.092	1.093	1.097	1.097	1.097
C_5–O_{13}	1.426	1.418	1.430	1.422	1.421	1.421
O_{13}–H_{14}	0.960	0.960	0.958	0.958	0.958	0.958
**Angles (degrees)**						
C_1=C_2-C_3	112.3	111.9	112.0	111.8	111.9	111.9
C_5-C_1=C_2	111.6	111.9	111.5	111.7	111.7	111.7
C_2-C_3-C_4	102.7	102.7	102.7	102.7	102.5	102.5
C_4-C_5-C_1	102.8	102.8	102.8	102.8	102.8	102.8
C_3-C_4-C_5	106.1	105.8	105.7	105.8	105.5	105.5
C_1-C_5-O_{13}	111.3	114.5	111.8	114.6	110.1	110.1
C_4-C_5-O_{13}	113.8	114.0	108.1	108.8	113.9	113.3
C_1=C_2-H_7	124.9	125.1	125.0	125.1	125.0	124.9
C_1-C_5-H_{12}	113.2	110.3	112.8	110.0	109.6	112.4
C_2=C_1-H_6	125.6	126.1	125.4	125.6	126.3	125.9
C_2-C_3-H_8	110.6	112.9	110.2	112.8	112.9	110.2
C_2-C_3-H_9	112.3	110.2	112.6	110.3	110.4	112.7
C_3-C_2-H_7	122.8	122.9	123.0	123.0	123.0	122.9
C_3-C_4-H_{10}	113.9	109.5	113.7	109.8	109.6	113.4
C_3-C_4-H_{11}	109.2	113.6	109.4	113.6	113.3	109.3
C_4-C_3-H_8	112.7	112.9	112.1	112.6	112.7	112.2
C_4-C_3-H_9	112.1	111.4	109.4	111.6	111.5	112.1
C_4-C_5-H_{12}	111.6	111.2	111.8	111.1	111.0	111.5

Table S1. *Continued*

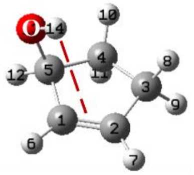

2CPOL Conformer

	A	B	C	D	E	F
**Angles (degrees)**						
C_5-O_{13}-H_{14}	107.1	107.2	108.0	108.1	107.9	107.6
C_5-C_4-H_{10}	110.7	107.4	110.6	106.4	106.6	111.4
C_5-C_4-H_{11}	109.0	112.8	109.0	112.8	113.2	108.9
C_5-C_1-H_6	122.5	122.0	123.0	122.6	121.9	122.4
H_8-C_3-H_9	106.6	106.8	107.1	106.9	106.9	107.1
H_{10}-C_4-H_{11}	108.0	107.6	108.3	108.2	108.4	108.0
H_{12}-C_5-O_{13}	104.5	104.3	109.3	109.3	109.3	109.4
**Angles between bonds**						
C_1=C_2 / O_{13}-H_{14}	127.3	108.9	44.0	-2.2	-123.8	-108.3
C_1=C_2 / C_3-C_4	-10.5	14.1	-12.6	13.6	14.0	-12.8
C_2=C_1 / C_5-C_4	15.1	-13.3	15.5	-14.1	-14.9	15.0
C_3-C_2 / C_1-C_5	-3.0	-0.5	-1.9	0.3	22.7	-1.4
C_2-C_3 / C_4-C_5	19.2	-21.4	21.4	-21.6	-179.4	21.2
C_5-C_1 / C_3-C_4	-13.6	13.9	-14.7	14.2	14.9	-14.5
C_1-C_5 / O_{13}-H_{14}	45.5	55.7	-54.7	-73.1	-171.8	-172.8
C_4-C_5 / O_{13}-H_{14}	-70.1	-62.3	-167.2	172.4	73.4	74.4
**Selected distances (Å)**						
C_1-H_{14}	2.471	2.593	2.560	2.741	3.219	3.194
C_2-H_{14}	3.028	3.490	3.544	3.953	4.196	3.992
Mid(C_1=C_2)-H_{14}	2.682	3.001	3.019	3.335	3.680	3.554
